# Primary Dural Repair in Minimally Invasive Spine Surgery

**DOI:** 10.1155/2013/876351

**Published:** 2013-05-30

**Authors:** Raqeeb M. Haque, Sohaib Z. Hashmi, Yousef Ahmed, Omar Uddin, Alfred T. Ogden, Richard Fessler

**Affiliations:** Department of Neurological Surgery, Feinberg School of Medicine and McGaw Medical Center, Northwestern University, Chicago, IL 60611, USA

## Abstract

We describe an effective surgical technique in primary repair of the spinal dura during minimally invasive spine surgery (MISS). *Objective*. Minimally invasive spine surgery includes the treatment of intradural lesions, and proper closure of the dura is necessary. However, primary dural closure can be difficult due to the restricted space of MIS retractors and the availability of appropriate surgical instrumentation. *Methods*. We describe the use of a needle already used in the pediatric neurosurgical arena that can facilitate easier and safer closure of spinal dura through MISS retractors in two illustrative intradural cases. *Results and Discussion*. The primary dural closure technique is described and patient demographics are included. The instruments specifically used for the intradural closure through MIS retractor systems include (1) 4-0 Surgilon braided nylon (Covidien, Dublin, Ireland) with a CV-20 taper 1/2 circle, 10 mm diameter needle; (2) Scanlan (Saint Paul, MN, USA) dura closure set. *Conclusion*. Successful primary dural repair can be performed on primary and incidental durotomies during minimally invasive spinal surgery. We describe the novel use of a 10 mm diameter needle to help surgeons safely and efficiently close the dura with more ease than previously described.

## 1. Introduction

Minimally invasive spine surgery continues to have an increasing role in the treatment and management of spinal disorders. Until very recently, open spine surgery remained the gold standard for the correction of spinal disorders; however, with the advancement of minimal approach techniques and technologies, the application of MISS continues to expand [1]. Initially applied in the treatment of lumbar spinal decompression, the current uses for MISS include management of spine deformity and spine tumors. As the literature confirming similar clinical outcomes between MISS and open surgery [2, 3] becomes available, MISS will be more widely adapted. 

Reduced anatomical exposure and limited working corridors necessitate novel surgical techniques in MISS to perform similar open procedures. Advancement of the overall applicability of MISS is largely dependent on technical feasibility of procedures. Excision and removal of intradural extramedullary tumors require dural incision. The incisional durotomy can be performed through tubular access; however, dural closure remains difficult. Additionally, the unintended disruption of the dura mater is a known complication associated with several spine surgery procedures. The reported incidence of IDs ranges from 1.1% to 17.4% [4]. We describe, through two illustrative cases, an effective and reproducible method for MISS primary dural closure using 10 mm tapered needle not used in the MIS setting before.

## 2. Methods

### 2.1. Illustrative Case 1

**Figure 1:**
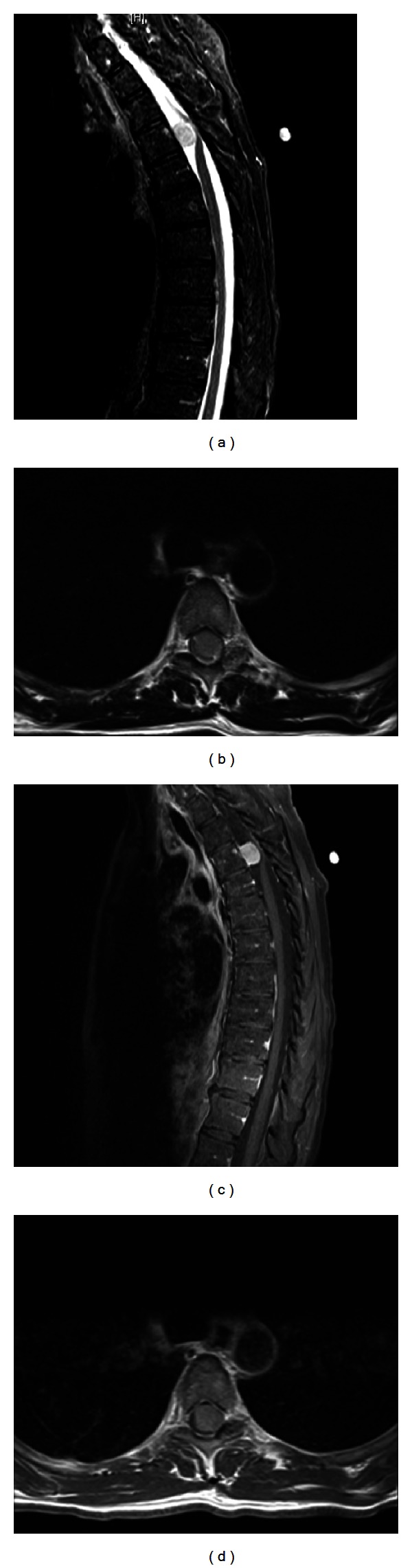
(a) Pre-op sagittal T2 MRI, meningioma at T4 level. (b) Pre-op axial T2 MRI, meningioma at T4 level. (c) Pre-op sagittal T1 MRI with contrast, meningioma at T4 level. (d) Pre-op axial T1 MRI with contrast, meningioma at T4 level.

A 56-year-old female presented to our institution with difficulty in balance, poor bilateral lower extremity coordination, and progressive radiculopathy spanning the left chest. On examination, the patient was not found to have sensory or motor deficits. Upon further diagnostic evaluation, MRI imaging revealed a T4 spinal cord mass (Figures 1(a)–1(d)). The ventral intradural extramedullary lesion was located eccentrically to the left side and demonstrated contralateral ventral compression of the spinal cord. After explaining the risks of the procedure, the patient elected to undergo minimally invasive resection of the intradural extramedullary mass.

The patient was placed in the prone position and underwent general endotracheal anesthesia. Preoperative fluoroscopic imaging was used to identify appropriate spinal level. A 1.5 cm incision left of midline centered over the left T4 hemilamina and transverse process was made. Metzenbaum scissors incised the fascia and blunt finger dissection was used to expose muscle and soft tissue surrounding the left T4 hemilamina. Muscular dilators and tubular retractor system were used to expose T3, T4, and T5 hemilaminas down to bone (a total depth of 3 centimeters). Residual amount of soft tissue remaining in the working channels was eliminated using Bovie cautery. After bony exposure, appropriate spinal level was confirmed with counting ribs and counting up from the sacrum with real-time intraoperative fluoroscopy.

**Figure 2:**
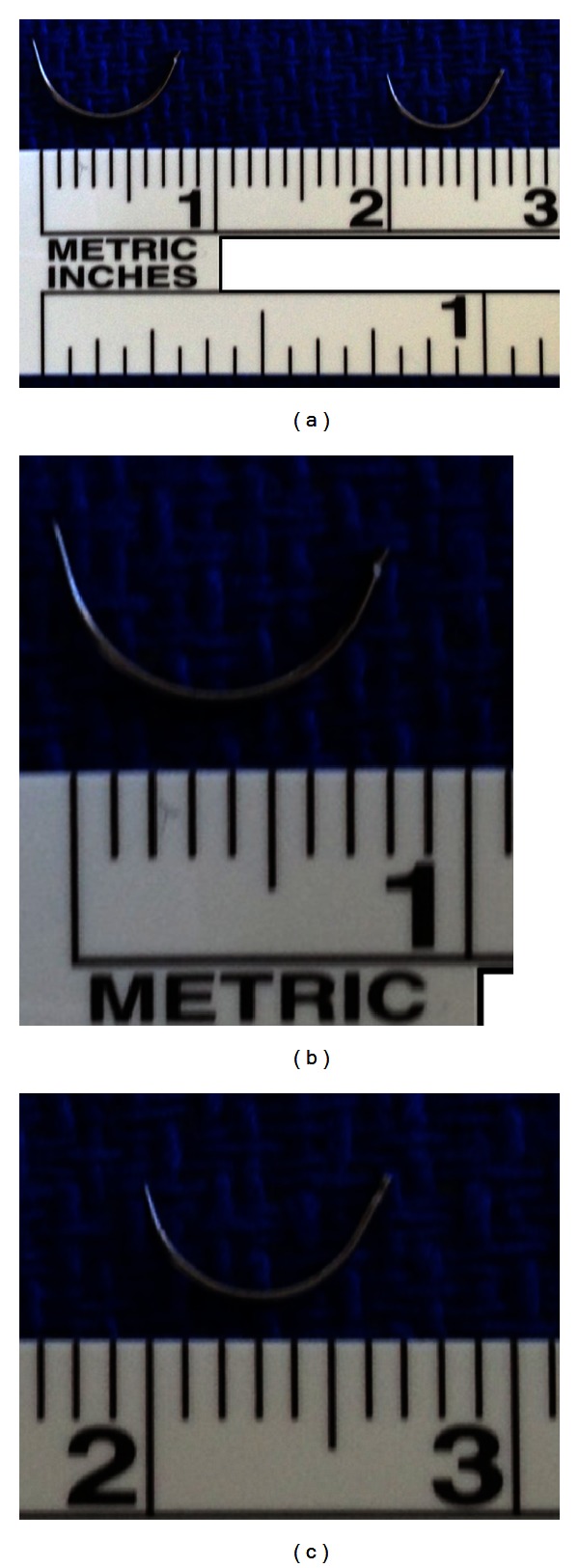
(a) CV-22 (right) and CV-20 (left) needles next to each other for comparison, (b) CV-22 taper needle is 1/2 circle with 13 mm diameter, and (c) CV-20 taper needle is 1/2 circle with 10 mm diameter.

**Figure 3:**
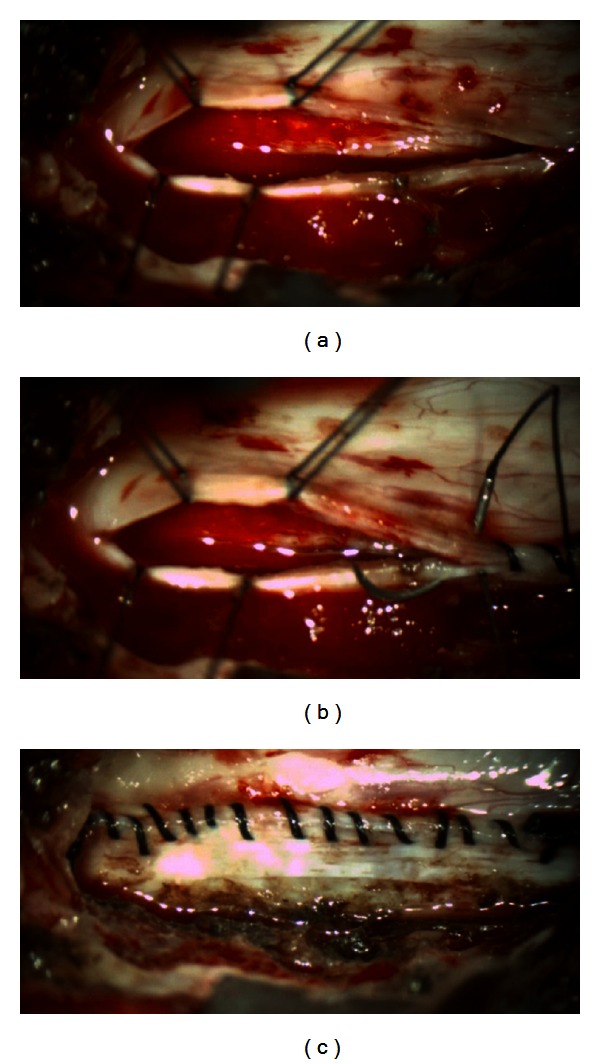
(a) View of the durotomy before dural closure. (b) Continuous, running midline closure of the dura was performed using a CV-20 needle with 4-0 Surgilon braided nylon. (c) Completion of dura closure.

**Figure 4:**
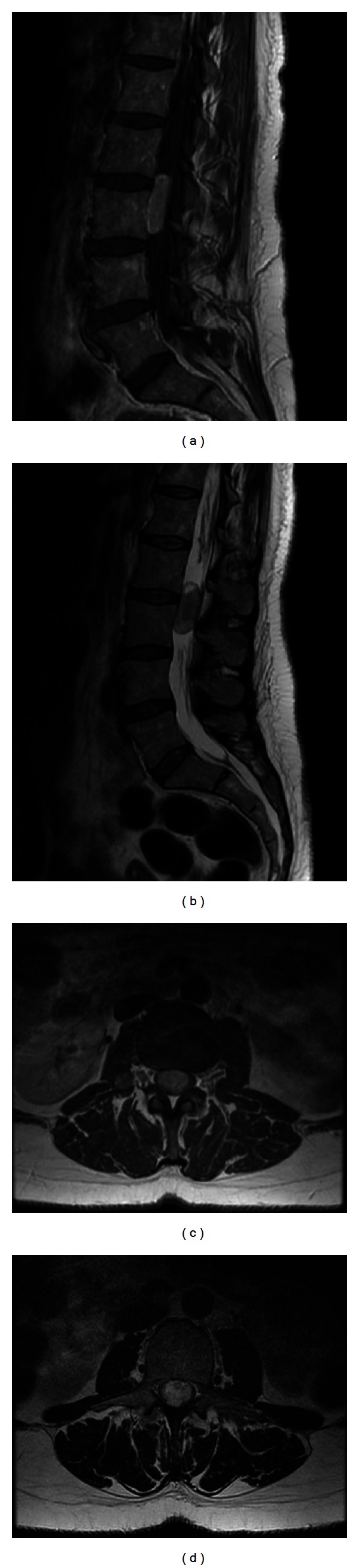
(a) Pre-op sagittal T1 with contrast MRI showing L2-L3 paraganglioma. (b) Pre-op sagittal T2 MRI showing L2-L3 paraganglioma. (c) Pre-op axial T1 with contrast MRI showing L2-L3 paraganglioma. (d) Pre-op axial T2 MRI showing L2-L3 paraganglioma.

Complete hemilaminectomy at the T4 level was performed. A drill was used to thin the left hemilamina and standard Kerrison punch was used to remove the hemilamina. The contralateral side was exposed by drilling the ventral hemilamina to the contralateral pedicle. Additionally, partial inferior T3 and partial superior T5 ipsilateral hemilaminectomies were performed using the same technique. Hemostasis was achieved using bipolar cautery as well as bone wax. With the use of the microscope, the dura was incised using an arachnoid knife. The dural edges reflected using 4-0 Nurolon suture (Ethicon, Somerville, NJ, USA). The lateral margin of the tumor was visualized, and a small specimen was sent for pathologic analysis revealing meningioma. Tumor debulking was performed using bipolar cautery and CUSA ultrasonic aspiration (Integra, Plainsboro, NJ, USA), while carefully protecting the tumor-spinal cord interface. Using bipolar cautery, ablation of the lateral dural margin of the tumor bed was performed. Hemostasis was primarily maintained using Surgifoam (Ethicon, Somerville, NJ, USA). Once the tumor was debulked in its entirety, copious irrigation of intradural contents was performed. Continuous midline closure of the dura was performed using a CV-20 needle (Covidien, Dublin, Ireland; Figures 2(a)–2(c)) and 4-0 Surgilon braided nylon (Covidien, Dublin, Ireland) in a running fashion. Minimally invasive dural closure instruments (Scanlan, Saint Paul, MN, USA) were used through working channels. Valsalva maneuver confirmed tight closure of the dura without evidence of CSF leakage through the suture line. Tisseel fibrin sealant (Baxter, Deerfield, IL, USA) was used as an adjunct to ensure closure over primary repair. Fascial and skin closure was performed in a continuous fashion (Figures 3(a)–3(c)). 

### 2.2. Illustrative Case 2

A 48-year-old female presented to our institution with bilateral lower extremity radiculopathy and low back pain. Her symptoms progressively worsened over the three years prior to surgery. Physical examination revealed no sensory or motor deficits. Upon further diagnostic evaluation, lumbar MRI imaging revealed a L2-3 intradural mass (Figures 4(a)–4(d)). The patient elected to undergo minimally invasive resection of the intradural extramedullary mass. The risks of the procedure were explained including infection, bleeding and need for transfusion, CSF leak, need for further surgery, incomplete resection of tumor, weakness, paralysis, and the concomitant risk of anesthesia. 

The patient was placed under general anesthesia and endotracheal intubation was performed. The patient was then positioned in the prone in a Wilson frame. Fluoroscopy was used to confirm L2-3 level interspace. Skin incision was made 1.5 cm right of midline, and hemostasis was maintained with bipolar cautery. Bipolar cautery allowed dissection and incision into the paraspinal fascia. Blunt finger dissection was used to split muscle tissue overlying L2-3 lamina. Muscular dilators and tubular retractor system were placed in the wound to expose tissue cephalad to caudad. Medial and lateral retractors were used to expose the right L2–4 lamina and facets. The remaining soft tissue in the working channel was removed with Bovie cautery. After bony exposure, appropriate spinal level was confirmed with counting ribs and counting up from the sacrum with real-time intraoperative fluoroscopy. 

 Once appropriate level was confirmed, an angled curette was used to define the sublaminar plane below L3-4. Kerrison punch was used to perform a hemilaminectomy of L3 and L2. This approach was extended to incorporate medial facetectomy to the medial border of the pedicles. Hemostasis was obtained using bone wax. Legend drill was then used to drill the ventral surface of the spinous process and contralateral lamina of L2-3 to the contralateral pedicles. Hemostasis in the lateral gutters was achieved using Surgicel (Ethicon, Somerville, NJ, USA) and Surgifoam (Ethicon, Somerville, NJ, USA). With appropriate bony exposure, the dura was incised caudad to cephalad from the mid-vertebral body of L3 to mid-vertebral body of L2. The dural edges were reflected using 4-0 Nurolon suture. Excellent visualization and exposure of the tumor was established in the surgical field using a hook to manipulate overlying nerves laterally. A significant vascular pedicle was cut after coagulation using cautery. A hook was used to manipulate the tumor between L2-3 nerve roots and remove the tumor from the dural canal. The tumor was then resected en bloc. Tumor sample was sent to pathology revealing paraganglioma. After the tumor removal, copious irrigation of intradural contents with warm saline was performed. Continuous, running midline closure of the dura was performed using a CV-20 needle (Covidien, Dublin, Ireland; Figures 3(a)–3(c)) with 4-0 Surgilon braided nylon (Covidien, Dublin, Ireland). Minimally invasive dural closure instruments (Scanlan, Saint Paul, MN, USA) were used through working channels. Valsalva maneuver confirmed tight closure of the dura without evidence of CSF leakage through the suture line. DuraSeal (Covidien, Dublin, Ireland) was used as an adjunct to ensure closure over primary repair. Fascial and skin closure was performed in a continuous fashion. 

## 3. Results 

This technical report describes the novel use of previously used needle in pediatric neurosurgery in the setting of MIS surgery, illustrating its use in two patients undergoing resection of intradural extramedullary tumors with primary dural closure. Patients underwent surgery between July and September 2012. Both patients were females with an average age of 52 years (range 48 to 56). Presenting symptoms included radiculopathy, low back pain, loss of balance, and poor coordination. Physical exam findings were unremarkable in both patients. The postoperative pathologic diagnosis in this series revealed meningioma and paraganglioma. The intradural extramedullary tumor locations included T4 thoracic and L2-3 lumbar regions. 

 Both patients underwent general endotracheal anesthesia for MISS procedure. Mean intraoperative time was 280 minutes (range 220 to 340 minutes). The mean estimated blood loss was 75 mL (range 50–100 mL). No complications were encountered intraoperatively or postoperatively. Neither patient required conversion to open exposure during surgery. As a result of reproducible and water-tight dural closure, patients were admitted postoperatively for only two days with ambulation beginning on postoperative day one for both patients. The ability to quickly mobilize the patient compared to an open case can be attributed to both the confidence in dural closure and MIS techniques. 

**Figure 5:**
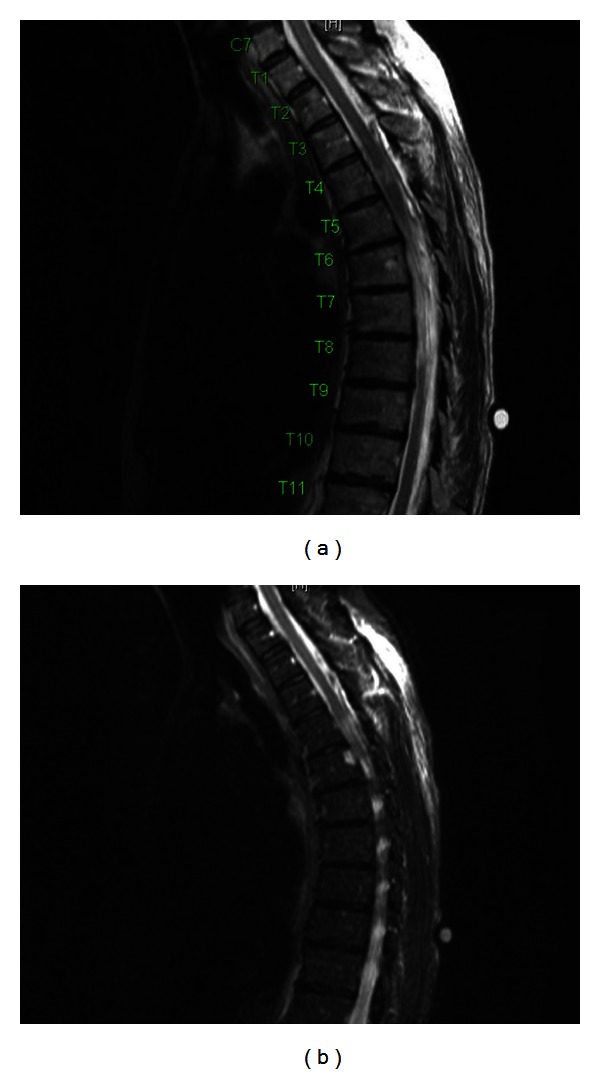
(a) Post-op sagittal T2 MRI with complete dural closure and complete tumor resection. (b) Post-op sagittal T2 MRI with complete dural closure and complete tumor resection.

 Postoperatively, patients were assessed at the postoperative 24 weeks with resolution of symptoms, wound intact, and no evidence of any spinal leak. Patient no. 2 post-op imaging showed complete resection of tumor at two days after operation (Figures 5(a) and 5(b)). Neither patient displayed CSF leak on MRI imaging. Both patients experienced complete relief of presenting symptoms at followup.

 Primary dural repair can present itself as a technically challenging aspect of MISS, especially when performed using established techniques. The narrow lumen of the tubular retractor restricts complete manipulation of larger, conventional needles such as the CV-22. With the novel use of the CV-20 needle, no cerebrospinal fluid leak was identified after primary dural repair. 

## 4. Discussion

Surgical treatment of primary spinal cord tumors often yields favorable results. These outcomes may in large part be due to the benign nature of the significant majority of these tumors. Up to 78% of all primary spinal cord tumors diagnosed are benign, while only 22% are found to be malignant [5]. Traditional open surgical exposure requires a posterior midline approach with muscle splitting and soft tissue dissection to access bony structures. Most often, bilateral laminectomy is required for proper exposure of the entire tumor and maximum resection. Several studies have proved the excellent outcomes with surgical management of intradural spinal cord tumors. Tumor locations may play a key role in outcomes success. Extramedullary and rostral location have been found to be a strong predictors of greater total tumor resection [6]. 

While open surgical exposure and technique for treatment of spinal cord tumors has been well established, the field of MISS has an increasing presence. MISS was originally limited to select indications, primarily used in decompression of lumbar spinal stenosis and lumbar discectomy. However with new advancements, MISS has expanded its applicability to the management of complex spinal pathology [1]. An increasing volume of evidence demonstrates the efficacy and advantages of MISS in the resection of intradural tumors [7, 8]. Recent literature shows shorter operative time, decreased intraoperative blood loss, increased preservation of structural bone and ligament anatomy, and decreased postoperative deformity rate of MISS hemilaminectomy compared to traditional open posterior midline laminectomy of cervical intradural extramedullary tumor resection [9]. 

 Several retrospective reviews show technical ability of complete resection of intradural extramedullary tumors through MISS [7–10]. However, certain procedural techniques demand further development. One primary limitation of MISS compared to open traditional surgery is the restricted working corridor. While MISS surgical dissection and removal of the tumor is largely consistent with open surgery, dural repair and closure must be adapted for a constrained surgical field. Primary dural closure through tubular access can be technically difficult. Alternative methods to dural closure have been employed including reapproximation with adjuvant biosynthetic sealants, the use of titanium clip closure, and dural closure with U-clip device (Medtronic, Minneapolis, MN, USA) without need for knot tying [7, 11]. 

 We have described a repeatable procedure for primary dural closure using instrumentation designed for MISS. Continuous running midline closure of the dura was accomplished with the use of CV-20 taper needle and 4-0 Surgilon braided nylon (Covidien, Dublin, Ireland) and minimally invasive dural closure instruments (Scanlan, Saint Paul, MN, USA) though working channels. Limitations to use of this technique include surgeon's skill as well as availability of proper instrumentation. However, with proper surgical training, MISS dural closure through a limited surgical field can be accomplished. The standard CV-22 needle previously used was 13 mm in diameter, making it more difficult to manipulate through the MIS retractors. The novel use of the CV-20 needle allows for maneuverability intraoperatively (Figure 3(b)). We found no evidence of CSF leak intraoperatively or postoperatively with MISS primary repair of the dura. Advancements in instrumentation specifically designed for MISS allow for greater adaptation and efficacy in reaching operative goals. It is our aim that this report of MISS primary dural closure in spinal tumor resection will allow adaptation of the technique, for both primary and incidental durotomies. As a result, a highly repeatable technique comparable to open surgical procedure may aid future clinical evidence investigating MISS outcomes. 

## 5. Conclusions

 Effective complete resection of intradural extramedullary tumors has been accomplished through minimally invasive spine surgery. However, dural repair can be challenging through a limited surgical exposure through working channels. This report describes an effective, repeatable technique of primary midline, running dural closure with the use of instrumentation designed for MISS. We hope that this report will make other MISS surgeons aware of the novel use of an already existing pediatric neurosurgical needle (CV-20) when performing primary dural closure within tubular retractor systems.
